# Local Delivery of Pirfenidone by PLA Implants Modifies Foreign Body Reaction and Prevents Fibrosis

**DOI:** 10.3390/biomedicines9080853

**Published:** 2021-07-21

**Authors:** Alexey Fayzullin, Semyon Churbanov, Natalia Ignatieva, Olga Zakharkina, Mark Tokarev, Daniil Mudryak, Yana Khristidis, Maxim Balyasin, Alexandr Kurkov, Elena N. Golubeva, Nadejda A. Aksenova, Tatyana Dyuzheva, Peter Timashev, Anna Guller, Anatoly Shekhter

**Affiliations:** 1 Department of Experimental Morphology and Biobanking, Institute for Regenerative Medicine, Sechenov First Moscow State Medical University (Sechenov University), 8-2 Trubetskaya st., 119991 Moscow, Russia; fayzullin_a_l@staff.sechenov.ru (A.F.); b.maxim4432@yandex.ru (M.B.); kurkov_a_v@staff.sechenov.ru (A.K.); shekhter_a_b@staff.sechenov.ru (A.S.); 2 World-Class Research Center “Digital Biodesign and Personalized Healthcare”, Sechenov First Moscow State Medical University (Sechenov University), 8-2 Trubetskaya st., 119991 Moscow, Russia; khristidis.yana@yandex.ru; 3 The Graduate School of Biomedical Engineering, University of New South Wales, Kensington, Sydney, NSW 2060, Australia; 4 The Faculty of Medicine, Health and Human Sciences, Macquarie University, Macquarie Park, Sydney, NSW 2060, Australia; 5 Department of Advanced Biomaterials, Institute for Regenerative Medicine, Sechenov First Moscow State Medical University (Sechenov University), 8-2 Trubetskaya st., 119991 Moscow, Russia; churbanov.semyon@gmail.com (S.C.); naksenova@mail.ru (N.A.A.); timashev_p_s@staff.sechenov.ru (P.T.); 6 Chemistry Department, Lomonosov Moscow State University, Leninskiye Gory 1-3, 119991 Moscow, Russia; nyu@kge.msu.ru (N.I.); legol@mail.ru (E.N.G.); 7 Institute of Photon Technologies, Federal Scientific Research Centre “Crystallography and Photonics” of Russian Academy of Sciences, 2 Pionerskaya st., Troitsk, 142190 Moscow, Russia; olzakharkina@gmail.com; 8 Sklifosovsky Institute for Clinical Medicine, Sechenov First Moscow State Medical University (Sechenov University), 8-2 Trubetskaya st., 119991 Moscow, Russia; tokmv9@gmail.com (M.T.); mdl.surg@gmail.com (D.M.); dtg679@gmail.com (T.D.); 9 Department of Polymers and Composites, N.N. Semenov Federal Research Center for Chemical Physics, 4 Kosygin st., 119991 Moscow, Russia; 10 Laboratory of Clinical Smart Nanotechnologies, Institute for Regenerative Medicine, Sechenov First Moscow State Medical University (Sechenov University), 8-2 Trubetskaya st., 119991 Moscow, Russia

**Keywords:** implantable drug delivery systems, pirfenidone, polylactic acid, polymer implants, foreign body reaction, fibrosis, peri-implant fibrosis, anti-fibrotic therapy, quantitative histopathology, collagen

## Abstract

Peri-implant fibrosis (PIF) increases the postsurgical risks after implantation and limits the efficacy of the implantable drug delivery systems (IDDS). Pirfenidone (PF) is an oral anti-fibrotic drug with a short (<3 h) circulation half-life and strong adverse side effects. In the current study, disk-shaped IDDS prototype combining polylactic acid (PLA) and PF, PLA@PF, with prolonged (~3 days) PF release (in vitro) was prepared. The effects of the PLA@PF implants on PIF were examined in the rabbit ear skin pocket model on postoperative days (POD) 30 and 60. Matching blank PLA implants (PLA^0^) and PLA^0^ with an equivalent single-dose PF injection performed on POD0 (PLA^0^+injPF) served as control. On POD30, the intergroup differences were observed in α-SMA, iNOS and arginase-1 expressions in PLA@PF and PLA^0^+injPF groups vs. PLA^0^. On POD60, PIF was significantly reduced in PLA@PF group. The peri-implant tissue thickness decreased (532 ± 98 μm vs. >1100 μm in control groups) approaching the intact derma thickness value (302 ± 15 μm). In PLA@PF group, the implant biodegradation developed faster, while arginase-1 expression was suppressed in comparison with other groups. This study proves the feasibility of the local control of fibrotic response on implants via modulation of foreign body reaction with slowly biodegradable PF-loaded IDDS.

## 1. Introduction

Implantation of biomaterials, prostheses, devices, implantable drug delivery systems (IDDS) [1] and other xenogeneic structures into the human and animal bodies is accompanied by a local immune response known as foreign body reaction (FBR) [2,3,4]. It starts from the formation of protein corona and adhesion of macrophages and other cells [5] on the implant surface, followed by fusion of the macrophages into foreign body giant cells (FBGC) and further progression of inflammation, angiogenesis and blood vessels maturation, followed by the fibrous capsule formation [4]. Acute phase of FBR inflammation contributes to the destruction (resorption, lysis) of the implant, while the later phase leads to its encapsulation by dense connective tissue capsule and isolation from the host tissue. The balance between the early, pro-inflammatory, lytic and the later, pro-fibrotic, FBR is orchestrated by the respective secretory switch (known as M1 and M2 polarization [6,7]) in the macrophage population that, to a great extent, depends on the surface properties of the implant [8,9,10,11,12]. The macrophage markers of such switch include, in particular, inducible nitric oxide synthase (iNOS) as a marker of acute inflammation/resorptive FBR and arginase-1 (Arg1) as a marker of pro-regenerative/fibrotic response [13,14]. Interestingly, the FBGC that form by the fusion of macrophages also can be classified by their surface markers and the secretome profile as M1- or M2-skewed cells [15]. However, it is important to emphasize that in vivo macrophages can have mixed phenotypes and co-express the M1 and M2 signatures, presenting rather a continuum of functional transformations [16].

The extent of the implant biodegradation depends on its material properties. This allows to classify the implantable materials and devices as fully or partially biodegradable, and non-biodegradable [1,17]. Implantation of non-biodegradable structures result in “frustration” of macrophages and formation of FBGC [5] that lead to chronic FBR and excessive fibrosis, while the biodegradable implants induce a sequence and a combination of both, the resorptive and fibrotic responses [5].

Implantation of slowly-biodegradable and non-biodegradable structures in 2–4 weeks results in the growth of immature tissue enriched with fibroblasts and blood capillaries (similar to granulation tissue in wounds) between the FBGC layer and the host resident tissue. This immature tissue is gradually replaced by fibrous connective tissue and becomes the peri-implant capsule [4]. The early-stage peri-implant capsules are enriched with blood vessels, fibroblasts, and immune cells [4]. Further maturation of the peri-implant capsule reflects peri-implant fibrosis (PIF) process [4]. While still significantly influenced by the inflammatory phase of FBR and, in particular, by M1-skewed macrophages [18], PIF is strongly regulated by the transforming growth factor-beta1 (TGF-β1) pathway [19,20]. It is associated with activation of fibroblasts, pericytes, and macrophages. Many of these cells express a myofibroblast-like phenotype (α-smooth muscle actin positive (α-SMA^+^)) and contribute to the excessive accumulation of extracellular matrix components and the contraction of the capsular tissue. 

The complications associated with FBR and PIF are a common problem in reconstructive surgery and regenerative medicine [21,22,23]. Scarring and contractures of the peri-implant tissue are among the most common and poorly controllable post-implantation issues, including the adverse reactions associated with implantation of drug-eluting devices [24]. One of the proposed solutions to this problem is application of IDDS that provide local delivery of compounds able to modulate the FBR [20,25,26,27]. In this regard, the most known approach implies delivery of nonsteroidal anti-inflammatory drugs, glucocorticoids, tyrosine kinases inhibitors, angiogenic stimulators and other classes of drugs [20,24,28]. 

The range of FDA-approved drugs with the proven antifibrotic effect is currently limited to the TGF-β1 antagonist pirfenidone (PF) and an angiokinase inhibitor nintedanib. Both drugs are used in the treatment of idiopathic pulmonary fibrosis. The TGF-β1 blocking activity of PF [29] as well as the major role of TGF-β1 in various fibrotic pathologies position this drug as a leading anti-fibrotic agent. It has been demonstrated that PF suppresses myofibroblast transdifferentiation of fibroblasts, and downregulates macrophage polarization towards pro-fibrotic M2 phenotype, resulting in reduced collagen accumulation and employment of other anti-fibrotic mechanisms [30]. Importantly, PF acts not only on TGF-β1 effects associated with M2-polarized macrophages, but also suppresses the synthesis of pro-inflammatory cytokine, TNF-α [31,32] that is characteristic of M1 macrophages. Then, pharmacologically, PF is classified as immunosuppressant, with not only anti-fibrotic, but also anti-inflammatory, antioxidant [33] and phototoxic [34] activity effects. The off-label therapeutic potential of PF (beyond the idiopathic lung fibrosis) attracts significant attention. The promising effects of PF were demonstrated in clinical and experimental treatment of burns [35], liver fibrosis [36], renal interstitial fibrosis associated with chronic kidney allograft rejection [37], diabetic foot ulcers [38], glaucoma [39,40], multiple sclerosis [41], several types of malignant tumors [42,43,44,45,46,47,48,49] and other conditions.

However, despite the proven effectiveness of PF against TGF-β1 hyperactivation, there are serious obstacles for the wider application of this drug in the treatment of organ-specific fibroses. Firstly, the ubiquitous nature of the TGF-β pathway implies that its physiological role in reparation and normal morphogenesis may be affected by systemically applied PF. Another problem stems from the pharmacokinetic properties and side effects of PF that is applied as oral pills with the dose increasing during the treatment course and induces gastrointestinal complications and skin photo-irritation. The circulation half-time of PF is less than 3 h. PF also has a strong affinity to albumen and rapidly excreted from the body [50]. Phototoxicity of PF is an additional problem associated with its systemic application [34]. These conditions, plus frequency of oral dosing and the cost of the treatment, indicate that the local delivery and sustained/prolonged release of PF may help to significantly improve the efficiency and safety profile of PF [36,51,52,53,54].

Topically applied gel containing PF was shown to be effective in pediatric hypertrophic scars [55]. Orally administered PF decreased the PIF in a small-scale study of mammary augmentation with silicone implants in rats in 56 days after implantation [56]. A more recent study revealed an anti-scarring effect of intrableb PF injection performed following an implantation of anti-glaucoma drainage device and indicating the perspective of the locally administered PF for the control of FBR [30]. However, topical administration is not enough to respond to the challenge of PF short circulation time and rapid drop of the active dose. One of the possible solutions of this issue may be based on the development of PF-loaded IDDS. Various design principles can be used in development of such IDDS. We think that the application of polymer IDDS for PF delivery may be among the most feasible and flexible approaches because of the high controllability of the composition and the material properties of medical polymers, availability of the clinically approved slowly-biodegradable materials such as polylactic acid (PLA) and its co-polymers (reviewed in details in [57] and [58]), and the technologies for manufacturing of implants from these compounds [1,17]. A challenge in the polymer IDDS approach is the hydrophobicity of the PLA-based polymers [58], that limits the loading capacity of such implants for highly hydrophilic drugs (like PF [54]).

In this study, we aimed to explore the effects of the PF-loaded slowly-biodegradable PLA implants (PLA@PF) acting as a prototype IDDS on FBR and PIF in a rabbit ear skin pocket model. The full-thickness excision skin wounds on the ventral side of the rabbit ears are intrinsically ischemic and conventionally used for modeling of scarring [59,60]. We slightly modified the excision models to secure precise implants’ positions, while preserving the pro-fibrotic environment around them. To bypass the loading capacity issue mentioned above, we applied an innovative approach of co-foaming of the medical grade PLA and PF powders in supercritical CO_2_ followed by the layer-by-layer laser sintering in a mode that allowed partial melting of the polymer granules but preserved the intact drug molecule structure. The key hypothesis of this study was that the PLA@PF can provide the prolonged local delivery of PF and improve the PIF outcome by modulation of FBR. “The reported results expand the state-of-the-art knowledge about the IDDS technologies, antifibrotic strategies in control of FBR to implanted biomaterials and the mechanisms of PIF.”

## 2. Materials and Methods

### 2.1. Preparation of Implants

Poly(DL-lactide) (PLA) polymer granules PURASORB PDL04 (№1824008, Purac, Netherlands) and PF (5-methyl-1-phenyl-2-[1H]-pyridone, HY-B0673, MedChemExpress, China) were used as primary materials for creating the implants. PLA granules (2–3 mm in diameter) were ground in a knife mill and put through a sieve mesh for a particle size of <100 μm. The obtained polymer powder was used for preparation of the implants used in the control groups (PLA^0^ and PLA^0^+injPF). Next, the polymer powder was mixed with a weighed portion of PF at the ratio of 500 μg of PF per 0.3 g of polymer. The resulting mixture was foamed in supercritical carbon dioxide (scCO_2_) as described previously [61] with minor modifications. In particular, the process was performed at 45 °C and 20 MPa pressure for 2 h. 

The obtained co-foamed PLA and PF material and the blank PLA powder were milled separately, sieved for the second time, and used to form the disk-shaped (10 mm in diameter and 1 mm in height) PLA@PF and PLA^0^ implants, respectively. The formation of implants was carried out by the method of surface-selective laser sintering described in our previous works [62,63] with minor modifications. Briefly, the powders were laser-treated by infrared fiber laser scalpel-coagulator LS 1.9 (IPG IRE-Polus, Fryazino, Moscow Oblast, Russia) with a wavelength of 1.94 microns under a power of 150 mW, and with a scanning speed of 15 mm/s to allow local sintering of the polymer surfaces only (<2 μm deep from the surface of polymer particles) without exerting significant thermal loads on the bulk of the polymer. The temperature control of sintering was carried out using a FLIR T530/24° infrared camera (FLIR Systems, Wilsonville, OR, USA). The temperature load on the bulk of the polymer did not exceed 63.2 °C. Since the melting point of PF was evaluated at 102–111 °C, the chemical structure of the drug impregnated into the implant was preserved [54].

### 2.2. Characterization of Implants

The surface texture of the sintered implants was studied using a scanning electron microscope (SEM) Phenom ProX (Phenom World, Eindhoven, The Netherlands) at an accelerating voltage of 10 kV. The size of the implants-forming particles’ was analyzed by segmentation and morphometry procedure on the SEM images using the ImageJ program, as described elsewhere [64]. The number of the measured particles was ≥1500. 

The estimation of contact angles was performed using the sessile drop method on a system for measuring surface properties (Acam D-2, Apex Instruments Co. Pvt. Ltd., Kolkata, India). 

In order to examine the uniformity of the distribution of PF in the polymer implant at the microlevel, we used the spin probe method [65]. For this purpose, we used a stable paramagnetic radical 4-Hydroxy-2,2,6,6-tetramethylpiperidine 1-oxyl benzoate (4-Hydroxy-TEMPO benzoate, #371343, Sigma-Aldrich), which resembles PF in chemical structure and geometric parameters. The spin probe was introduced into the PLA powder under conditions similar to those used for the preparation of PLA@PF powder (in scCO_2_ at 45 °C temperature and 20 MPa pressure). Electron paramagnetic resonance (EPR) spectra of 4-Hydroxy-TEMPO benzoate were recorded using a Bruker EMX 500 Plus X-band radio spectrometer at 90 K. The obtained samples of PLA-4-Hydroxy-TEMPO benzoate co-foamed powder were placed in quartz ampoules with an inner diameter of 3–4 mm. The spectra were recorded at a microwave power of 0.1 mW and a modulation amplitude of 0.1 mT. 

### 2.3. Drug Release and Entrapment Efficiency Analysis

The dynamics of the release of PF was compared for the composite PLA@PF powder and for the sintered PLA@PF implants of the same dry weight. The amounts of released PF were analyzed spectrophotometrically at a wavelength of 340 nm corresponding to the absorption profile of PF [66], with the samples being kept at 37 °C in phosphate buffer saline (PBS) pH 7.4 for 6 days. 

The entrapment efficiency of the PF in PLA@PF powder was calculated using the Equation (1):

(m1 − m2)/m1 × 100%(1)

where m1 and m2 are the masses of the initially added PF and non-entrapped PF, respectively.

### 2.4. The Surgical Procedures

The experiment in six chinchilla rabbits (males, 2–2.5 kg) was approved by the Local Ethical Committee of Sechenov University (Protocol #06-19/15.05.2019). The rabbits were kept under the standard vivarium conditions, one animal per cage and provided with complex granulated laboratory chow and constant access to water. 

For the surgery, the animals were anaesthetized by intramuscular injection of a solution of ZOLETIL 100 (VIRBAC, France; 6 mg/per 1 kg of animal body weight), supplemented with local anesthesia of the operating field with a solution of Novocain 0.5 %. The skin pockets (1.5 × 1.5 cm were formed on the ventral side of rabbit ears by blunt separation of skin derma from the perichondrium of the cartilage plate. Implants were surgically fixed subcutaneously with 3-0 Prolene (Ethicon, Bridgewater, NJ, USA) in the skin pockets to model peri-implant fibrosis. One control PLA^0^ implant, one PLA^0^ implant followed by injection of 500 μg of PF in 200 μm of PBS (PLA^0^+injPF) and one PLA implant loaded with 500 μg of PF (PLA@PF) were implanted in each ear. The implants were placed at a distance of at least 1.5 cm from the marginal ear artery and from each other. Postoperative antibacterial therapy was carried out by intramuscular injections of Baytril 5% (Bayer, Germany), the dose of 5 mg of Enrofloxacin per 1 kg of animal body weight, daily for five days after surgery. 

On the 30th and 60th postoperative days (POD30 and POD60, respectively), the rabbits were euthanized by the injection of a solution of ZOLETIL 100 (VIRBAC, Carros, France; 60 mg/kg of animal body weight). 

The sites of implantation were dissected together with the surrounding tissues at approximately 2–3 mm from the original wounds’ edges together with the implanted materials. Each of the dissected samples was divided into two parts: a half of each sample was fixed in 10% neutral buffered formalin; a third of the original sample was immersed in an O.C.T. cryogel (Fisher Healthcare, Pittsburgh, PA, USA) and snap-frozen in liquid nitrogen for cryobanking. In the remaining fragment of each sample, the peri-implant tissues (together with implant residuals) were mechanically separated from the surrounding skin and placed in cold sterile physiological saline solution for further thermal assays. The matching intact rabbit ear skin fragments were obtained from the unrelated experiment (as shared tissues). These samples of intact skin served as controls.

### 2.5. Histology

The implant material was dissolved during the standard histological processing of the samples performed for the paraffin embedding. Four-μm-thick sections of the formalin-fixed-paraffin-embedded tissue samples were stained with hematoxylin and eosin (H&E), Weigert-Van Gieson kit (VG) and with Picrosirius red (PSR) for the detection of collagen fibers. A LEICA DM4000 B LED microscope, equipped with a LEICA DFC7000 T digital camera running under the LAS V4.8 software (Leica Microsystems, Wetzlar, Germany) was used for the examination and imaging of the samples. The specimens were studied using conventional (for H&E, VG and PSR stained samples) and polarized light (PSR stained samples) microscopy.

### 2.6. Immunohistochemistry (IHC)

Four-μm-thick sections of the formalin-fixed-paraffin-embedded tissue samples were deparaffinized, incubated with 3% H_2_O_2_ for 10 min, underwent heat induced epitope retrieval (pH 6.0 sodium citrate buffer, 30 min in 80 °C water bath), additionally blocked with Background Block (Cell Marque, Rocklin, CA, USA) and incubated separately with mouse monoclonal primary antibodies against α-smooth muscle actin (α-SMA) (A2547, Merck, US, diluted 1:400), inducible nitric oxide synthase (iNOS) (MA5-17139, Invitrogen, US, diluted 1:400), or arginase-1 (Arg1) (ab239731, Abcam, UK, diluted 1:200) and detected by HRP-conjugated secondary goat antibodies (G-21040, Invitrogen, US, diluted 1:1000) and diaminobenzidine (DAB) with hematoxylin counterstaining. 

### 2.7. Morphometry

The peri-implant tissue thickness was measured in each histological sample at five sites located ~400 μm apart from each other at the center of the implantation site. The measurements were conducted from the interior surface of the dermal–epidermal junction to the upper surface of the perichondrium of the cartilage plate. 

The relative area of implants was measured in central parts of each histological slide by selection and segmentation of the white pixel areas with ImageJ software, calibrated in μm^2^, divided to the area of the cross-section of the intact implants of the corresponding length, that equals 3.5 × 10^5^ μm^2^ (considering that the original height of the implants was 1 mm, the diameter was 10 mm; and the average length of the intact implant fragment visible at the selected magnification of the microscope was 350 μm), and multiplied by 100%. 

The expression of α-SMA, iNOS and Arg1 was evaluated in the whole peri-implant complex. The evaluation of the staining (brown color of DAB) intensity was performed by semiquantitative scoring (Table 1).

### 2.8. Thermal Analysis 

Before starting thermal analysis, each sample was mechanically divided into three parts: a full-thickness sample (to obtain relevant thermograms) and separated peri-implant and subepidermal fragments of implantation sites (for spatial attribution of the endothermic peaks). Tissue samples of approximately 7–11 mg were excised, blotted with tissue paper to remove surface water, and placed in hermetically-sealed aluminum pans. Differential scanning calorimetry (DSC) measurements were performed using a Phoenix DSC 204 (Netzsch, Selb, Germany) differential scanning calorimeter with heating from 20 °C to 90 °C at the scanning rate of 10 °C/min. The resulting DSC curves were analyzed using Proteus® Thermal Analysis software. The heat of collagen denaturation was normalized to dry weight of the tissue. Deconvolution of the data in the 40–85 °C region into Gaussian peaks was performed by multi-peaks fitting using Origin 8.0 software. The fraction of the corresponding collagen population in the mixture was estimated via the area under each peak (estimated by peak deconvolution).

### 2.9. Statistical Analysis

The statistical analysis of the experimental quantitative data was performed with a standard program package GraphPad Prism version 8.00 for Windows (GraphPad Software, Inc., San Diego, CA, USA) and SPSS 26.0 (IBM, Armonk, NY, USA). The normal distribution of the quantitative data was checked by Shapiro–Wilk’s normality test. The intergroup differences were analyzed by the one-way ANOVA method followed by Tukey’s multiple comparison test. The search for the differences of the histological scores was conducted using Kruskal–Wallis test followed by Dunn’s multiple comparison test. Non-parametric analysis of intergroup comparisons was performed using Mann–Whitney U test. Analysis of correlations between the studied variables was performed using nonparametric Spearman’s correlation coefficient (Rs) and two-tailed statistical tests were applied. The statistical analysis results were presented as scatter plot graphs of the mean values and standard deviations (SD). *p*-Values equal or less than 0.05 were considered statistically significant.

## 3. Results

### 3.1. Characterization of Implants

**Figure 1:**
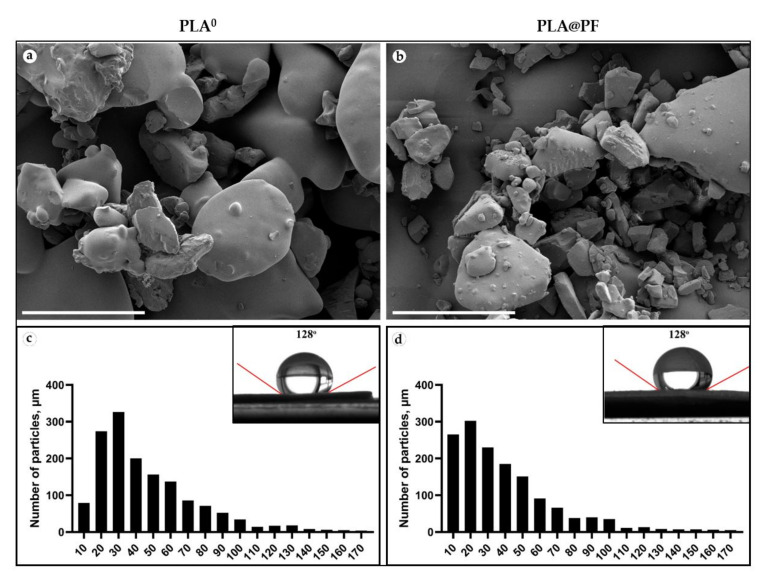
(**a**,**b**) SEM of the surface texture of PLA^0^ (**a**) and PLA@PF (**b**) implants. Scale bars 100 μm. (**c**,**d**) The histograms of the size distribution of 1500 randomly measured particles and measurements of the contact angles (inserts) of the PLA^0^ (**c**) and PLA@PF (**d**) implants.

**Figure A1:**
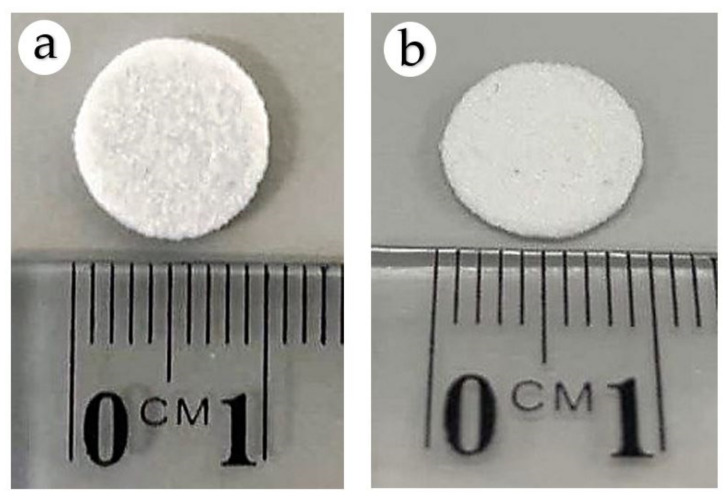
Optical image of PLA^0^ (**a**) and PLA@PF (**b**) implants.

Disk-shaped (1 mm in thickness and 10 mm in diameter) PLA^0^ and PLA@PF (Figure A1, Appendix A) implants were mechanically stable. The laser sintering regime provided only partial melting of polymer particles (Figure 1a,b). The average diameter of the particles located on the surface of the implants was 46.4 ± 29.4 μm (PLA^0^) and 40.0 ± 29.9 μm (PLA@PF) (Figure 1c,d and Table A1, Appendix A). According to the results of Mann–Whitney statistical test, the size of the surface-associated particles in PLA@PF implant was smaller than in PLA^0^ implant (*p* < 0.001), while it was varying in a similar range (see Table A1, Appendix A). The static contact angles of the sintered samples were 128 ± 3° for both PLA@PF and PLA^0^ implants (Figure 1e,f), signifying the similar hydrophobicity of the materials.

**Figure A2:**
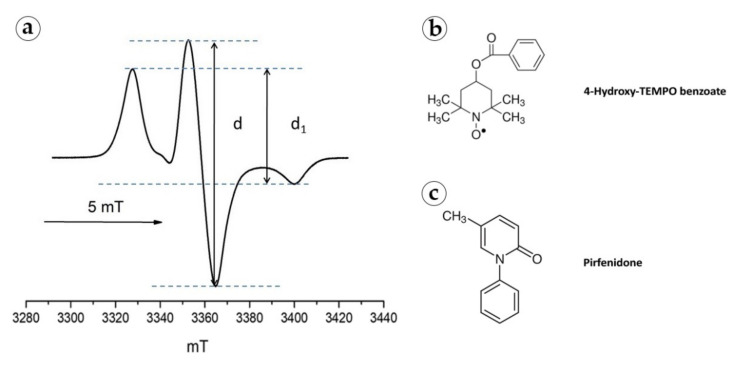
(**a**) EPR spectrum of 4-Hydroxy-TEMPO benzoate in a PLA implant at 90 K. Structural formulas of 4-Hydroxy-TEMPO benzoate (**b**) and PF (**c**).

Based on the analysis of the shape of the EPR spectrum (d1/d, where d1 is the distance between the maxima of the lateral components, and d is the central component amplitude, registered at 90 K in the absence of rotational mobility), the distances between 4-Hydroxy-TEMPO benzoate molecules within the PLA carrier were no less than 3 nm. In addition, there were no signs of a singlet line indicating local accumulation of the probe (Figure A2, Appendix A). Both facts indicated a uniform distribution of paramagnetic centers in the structure of the PLA implant loaded with 4-Hydroxy-TEMPO benzoate.

**Figure 2:**
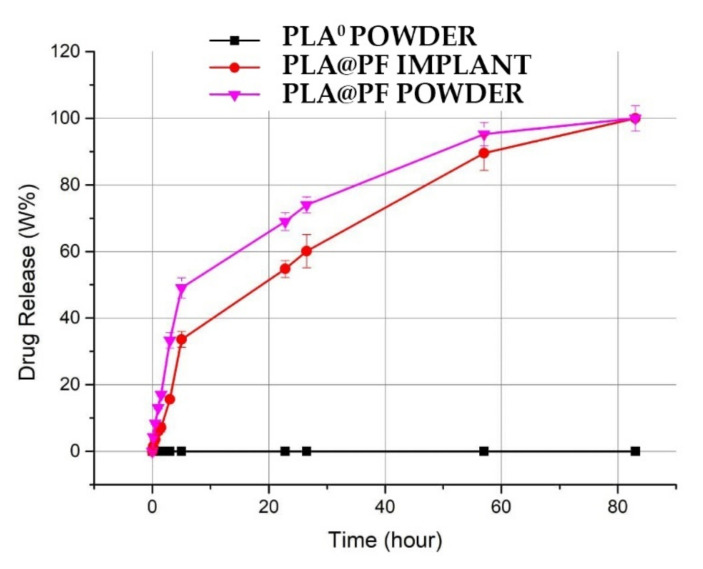
Spectrophotometric kinetics of cumulative amount PF release into PBS solution from the PLA@PF powder and PLA@PF laser-sintered implants (*n* = 3 per group) at 37 °C.

The achieved PF entrapment efficiency for PLA@PF implants was 97.9 ± 13.8%. The release of PF from the PLA@PF powder in PBS at 37 °C was very intense during the first hours and reached 49.1 ± 2.1% after 5 h of incubation (Figure 2). In contrast, PF release from the laser-sintered PLA@PF implant of the same mass was steadier, with only 33.6 ± 1.8% of the drug released over a 5 h interval. Total (100%) release of PF from both kinds of samples occurred in ~80 h.

### 3.2. Histology, Immunohistochemistry and Morphometry

#### 3.2.1. The Overall Structure of the Samples

**Figure A3:**
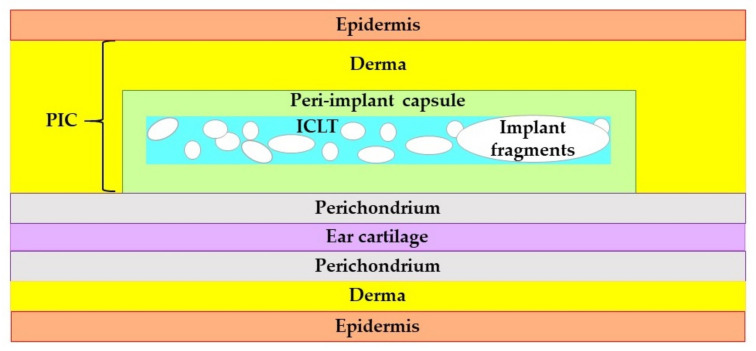
The schematic representation of the structure and positioning of the PIC in the rabbit ear skin pocket. Unscaled.

The excised tissue samples, generally, had the same structural components. The tissue elements of interest were located between the epidermal–dermal junction and the perichondrium of the ear cartilage (Figure A3, Appendix A). As the artificial (implant material) and natural (resident and formed de novo tissue) components were tightly interconnected, we termed this zone as peri-implant complex (PIC).

First, there were the residuals of the implant material that appeared on the tissue sections not as a bulk structure, but as empty “voids” due to the removal of PLA during the histological processing. The implants’ residuals were observed in all the samples, until the end of the experiment. However, their amount varied in different groups.

Next, the layers or patches of FBGC and varying amounts of loose connective tissue or organizing granulation tissue surrounded the implants’ residuals. This implant-colocalized tissue (ICLT) was easily identifiable on the sections stained with VG kit by picrinofilic color (yellow to orange) and on the samples stained with PSR and examined under bright light microscope (pale red color). 

The ICLT was encircled by the connective tissue peri-implant capsule that merged with the reticular layer of the skin derma on one side and bordered with the perichondrium on the other side. 

At the same time, the internal organization of the PIC, and the histomorphological features of FBR and PIF in the studied groups differed notably.

#### 3.2.2. The Morphometry of the PIC

**Figure 3:**
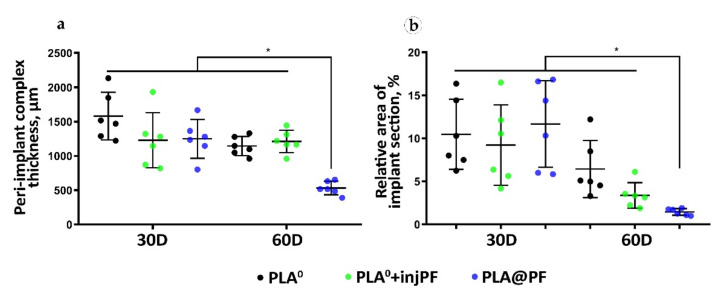
Morphometry of the PIC structure. (**a**) The total thickness of the PIC, μm. (**b**) Relative section area of the residual implant materials in the histological specimens, %. Mean ± SD, * *p* ≤ 0.05.

The thickness of the PIC. The PIC in the experimental groups was thicker than the intact skin derma (which had average thickness of 302 ± 15 μm) during the whole period of the experiment (Table 2 and Figure 3a). On POD30, there were no statistically significant differences between the studied groups in the PIC thickness (ANOVA, *p* = 0.180). Interestingly, from POD30 to POD60, the thickness of the PIC decreased only in PLA^0^ and PLA@PF groups, with statistical significance (Mann–Whitney test, *p* = 0.016 and *p* = 0.004, respectively). In contrast, in PLA^0^+injPF group, the thickness of the PIC did not change during the study period (*p* = 0.748). On POD60, the thickness of the PIC in PLA@PF group was statistically significantly lower than in PLA^0^ and PLA^0^+injPF (*p* < 0.001 in both cases). At the same time, there was no statistical significance in the difference in the thickness of PIC in PLA^0^ and PLA^0^+injPF groups (*p* = 1.000). The PIC in PLA@PF group at the end of the experimental period (POD60) was less than two times thicker than the intact skin derma; the difference still was statistically significant (*p* = 0.018). In control groups on POD60, the PICs were 4–5 times thicker than the intact derma. 

The relative area of the implant material on histological sections (Figure 3b, Table A2, Appendix A) did not statistically differ between the studied groups on POD30 (ANOVA, *p* = 0.663). In addition, there was no statistically significant change of the relative implant area in PLA^0^ group between POD30 and POD60 (*p* = 0.089). In contrast, in PLA^0^+injPF and PLA@PF groups, on POD60, the amount of implant material decreased, in comparison to the previous timepoint (*p* = 0.015 and *p* = 0.001, respectively). 

The most pronounced biodegradation (as measured on POD60) was observed in PLA@PF group, where the relative implant area was only 14.5 ± 3.8% of the original implant size, while in the PLA^0^ and PLA^0^+injPF it was 64.4 ± 33.2% and 33.8 ± 14.9%, respectively. The differences were statistically significant between PLA@PF and PLA^0^ groups (*p* = 0.004) and between PLA@PF and PLA^0^+injPF groups (*p* = 0.012), but not between PLA^0^ and PLA^0^+injPF (*p* = 0.066) groups. Application of PF either as an injection, or as a component of the implant resulted in statistically significant decrease of relative implant area as measured on POD60, in comparison with the PLA^0^ group (*p* = 0.005). 

#### 3.2.3. The Structural Dynamics of the PIC

**Figure 4:**
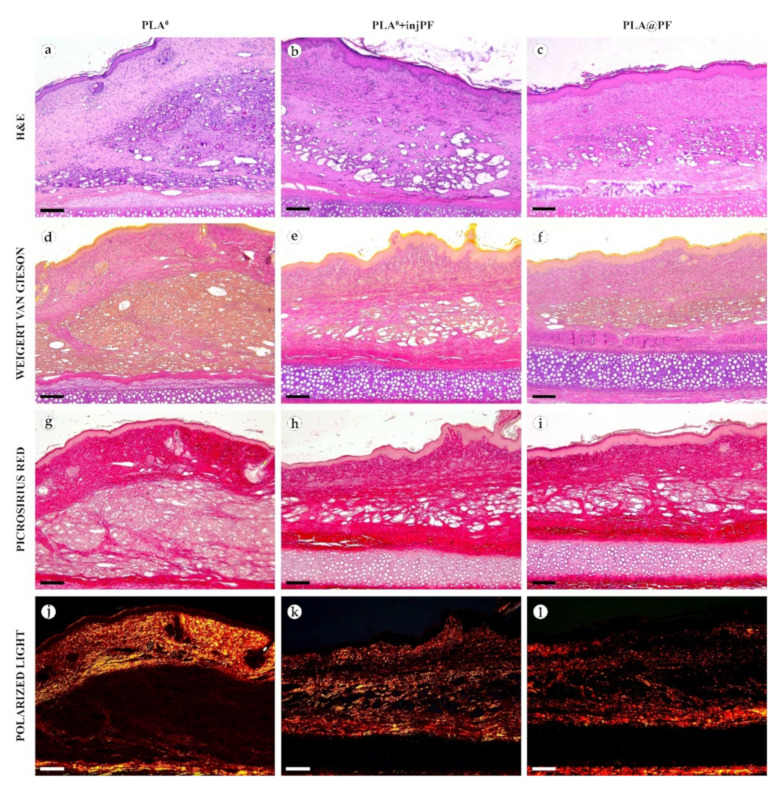
Histological examination of the peri-implant tissues on POD30, overview of the structure at a low magnification: H&E (**a**–**c**), VG (**d**–**f**) and PSR (**g**–**l**) staining, scale bar—200 μm, bright field (**a**–**i**) and polarized light (**j**–**l**) microscopies. The images of PSR stained samples taken by bright field and polarized light microscopy are location-matching. Columns depict the studied groups (PLA^0^, PLA^0^+injPF and PLA@PF implants).

**Figure 5:**
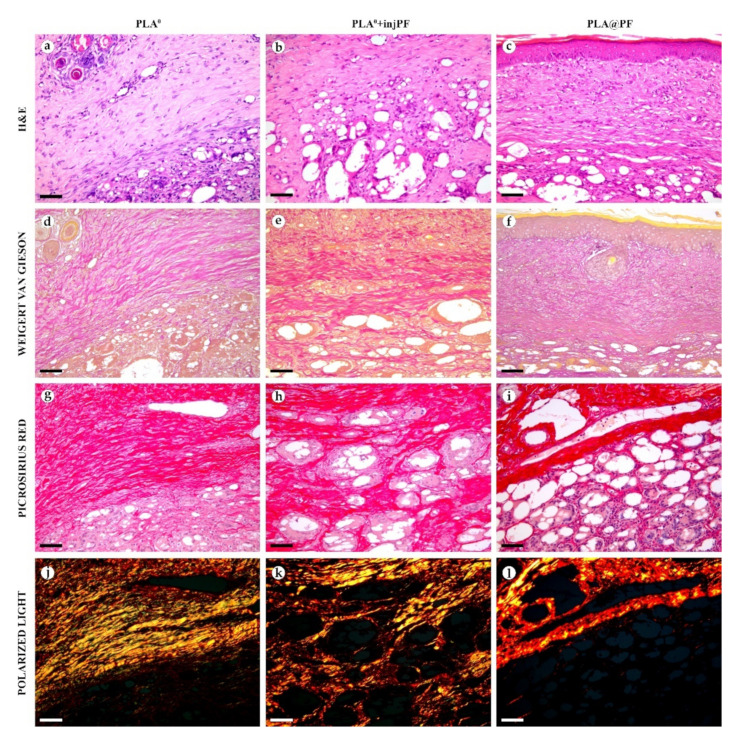
Histological examination of the peri-implant tissues on POD30, overview of the structure at a high magnification: H&E (**a**–**c**), VG (**d**–**f**) and PSR (**g**–**l**) staining, scale bar—50 μm, bright field (**a**–**i**) and polarized light (**j**–**l**) microscopies. The images of PSR stained samples taken by bright field and polarized light microscopy are location-matching. Columns depict the studied groups (PLA^0^, PLA^0^+injPF and PLA@PF implants).

In PLA^0^ group, on POD30 (Figure 4 and Figure 5a,d,g,j), the PIC included dense fibrous connective tissue capsule with parallel collagen fibers. The capsule was about 200–400 μm in thickness, it was moderately fuchsinophilic when stained by VG method and had bright red color in the sections stained with PSR (Figure 4 and Figure 5d,g). It also showed strong birefringence in PSR-stained samples under polarized light (Figure 4 and Figure 5j). The outer part of the capsule contained numerous blood vessels of small and medium caliber. The implant material was surrounded by multinuclear FBGC. The spaces between the FBGC were massively infiltrated by lymphocytes and macrophages and contained multiple fibroblasts (all these cells are generally termed below as non-FBGC cells) and blood vessels that together formed immature connective tissue (see the supplementary morphometric results for these elements in Table A3 and Table A4 in Appendix A). In contrast to the peri-implant capsule, the ICLT stained picrinofilic by VG, pale red with PSR and did not have birefringence in PSR-stained samples in polarized light. The fragments of implant visible in the samples of this group on POD30 were mostly small (~30–50 μm in size).

**Figure 6:**
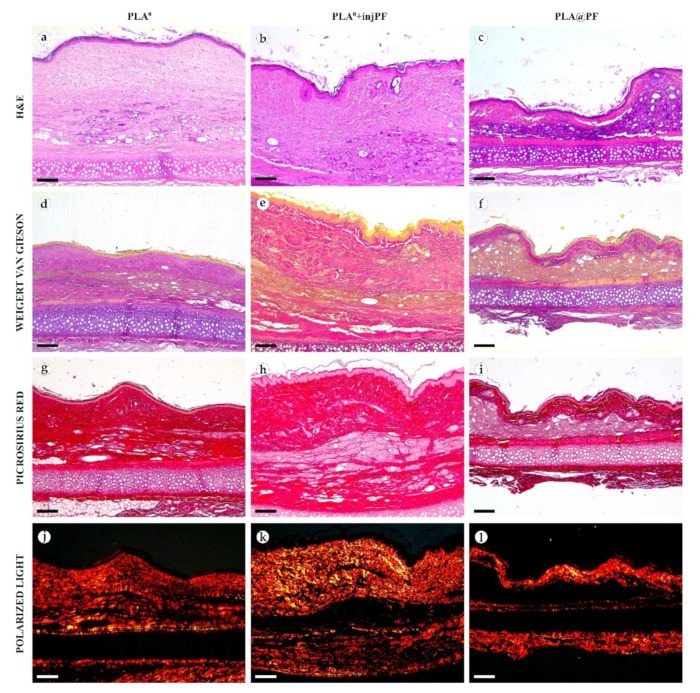
Histological examination of the peri-implant tissues on POD60, overview of the structure at a low magnification: H&E (**a**–**c**), VG (**d**–**f**) and PSR (**g**–**l**) staining, scale bar—200 μm, bright field (**a**–**i**) and polarized light (**j**–**l**) microscopies. The images of PSR stained samples taken by bright field and polarized light microscopy are location-matching. Columns depict the studied groups (PLA^0^, PLA^0^+injPF and PLA@PF implants).

**Figure 7:**
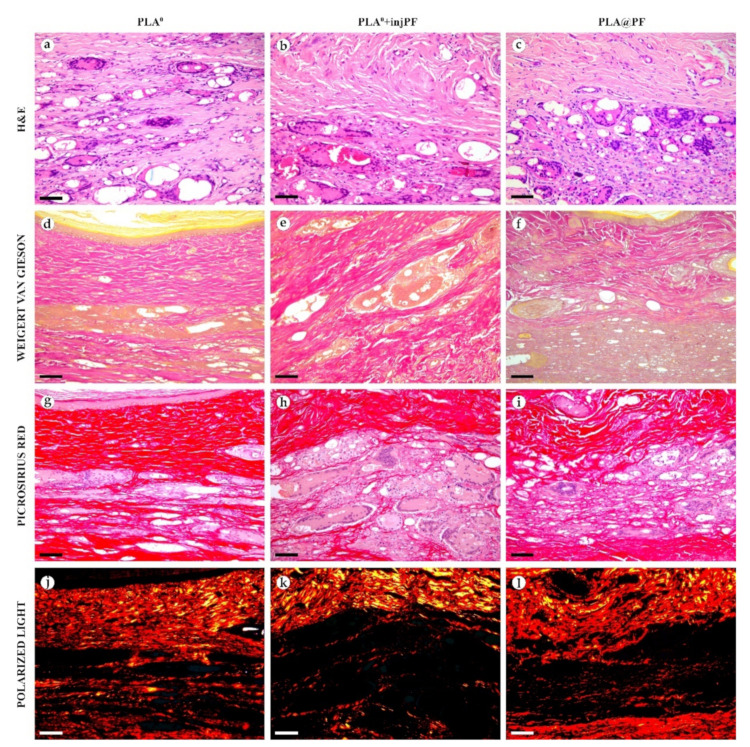
Histological examination of the peri-implant tissues on POD60, overview of the structure at a high magnification: H&E (**a**–**c**), VG (**d**–**f**) and PSR (**g**–**l**) staining, scale bar—50 μm, bright field (**a**–**i**) and polarized light (**j**–**l**) microscopies. The images of PSR stained samples taken by bright field and polarized light microscopy are location-matching. Columns depict the studied groups (PLA^0^, PLA^0^+injPF and PLA@PF implants).

On POD60, in PLA^0^ group (Figure 6 and Figure 7a,d,g,j), the peri-implant capsule merged with derma and was poorly identifiable. Its thickness was estimated to be ~200 μm. It had increased fuchsinophilia (in VG-stained samples), in comparison with POD30, vibrant red PSR staining and strong birefringence (PSR-polarized light microscopy), while the tinctorial properties did not change. The capsule contained fibroblasts and blood vessels of small and medium caliber. No foci of immune cells infiltrate, or signs of microcirculatory disorders were observed. The ICLT was separated in several parallel layers by immature connective tissue sheaths that were pale red when stained by VG, bright red with PSR staining and not birefringent by PSR staining examined under polarized light. The size of the implant particles was mostly small (<50 μm), but several large (~100 μm) aggregates of the particles were also visible.

In PLA^0^+injPF group, on POD30 (Figure 4 and Figure 5b,e,h,k) the peri-implant capsule had moderate density and contained polymorphous fibroblasts and immune cells (mostly lymphocytes). The overall thickness of the capsule varied between 200 and 400 μm. The capsular tissue did not present as an entire layer but formed separate parallel connective tissue sheaths in the inner parts of the capsule. The outer portion of the capsule was less vascularized than in PLA^0^ group. The ICLT contained mostly FBGC and small number of fibroblasts. It was stained similarly to the ICLT in PLA^0^ group. In several cases in PLA^0^+injPF group, the connective tissue of the capsule grew into the zone of the ICLT, dividing it into fragments. These ingrowths of the capsular connective tissue were fuchsinophilic (VG), bright red (PSR) and birefringent (PSR in polarized light). The fragments of implant in this group were mostly large (≥100 μm).

On POD60, in PLA^0^+injPF group (Figure 6 and Figure 7b,e,h,k) a fibrous peri-implant capsule had tinctorial similarity with the surrounding derma. The capsule visually tightly merged with the skin derma, in the same way as in PLA^0^ group on POD60. The capsule contained thick collagen fibers and bundles running parallel to the epidermis and splitting the ICLT into parallel blocks. These blocks were formed by small (<50 μm) fragments of implant material that were engulfed by the merged multinuclear FBGC. Fibroblasts, medium caliber vessels and a small number of immune cells were also observed in the ICLT. The tinctorial properties of this tissue remained the same as on POD30, while its volume visually decreased.

In PLA@PF group, on POD30 (Figure 4 and Figure 5c,f,i,l) the PIC included a thin (~100 μm) and dense peri-implant capsule with parallel oriented collagen fibers and elongated fibroblasts with strongly eosinophilic cytoplasm. The capsule separated the implant material and the ICLT containing FBGC from the intact skin and its appendages, hair follicles and glands. When stained by VG method, the capsule was slightly more fuchsinophilic, than in other groups on POD30. It also stained brighter red with PSR and was strongly birefringent (PSR in polarized light). The number of blood vessels around the implant was higher than in other two studied groups. The ICLT contained FBGC and rare foci of immune cells infiltration and looked like organizing granulation tissue by the number of capillaries and fibroblasts. The ICLT was picrinofilic (by VG staining), pale red when stained with PSR and non-birefringent (PSR-polarized light microscopy). No signs of massive connective tissue ingrowth from the side of peri-implant capsule were notable in the area of the ICLT. The fragments of the implant material surrounded by FBGC and granulation tissue in this group were smaller than the implant fragments separated by connective tissue ingrowth in PLA^0^+injPF group. The size of the implant particles was relatively regular, with the majority of them having a diameter of 50–80 μm.

On POD60, in PLA@PF group, (Figure 6 and Figure 7c,f,i,l)the implant was surrounded by organized connective tissue capsule, resembling native tissue of the rabbit ear dermis with very mild signs of fibrotic transformation. Medium caliber blood vessels were visible at the border of the capsule and derma and had no signs of circulatory disorders. The PLA@PF implant material was compact, and co-localized with the densely packed FBGC without interlayers of collagen fibers, but with fibroblasts and multiple lymphocytes. The implant fragments were mostly small, but rare aggregates (≥100 μm) were present. The tinctorial properties of the PIC components were similar to those observed on POD30 in the same group.

#### 3.2.4. The Immunohistochemical Examination of the PIC

**Figure 8:**
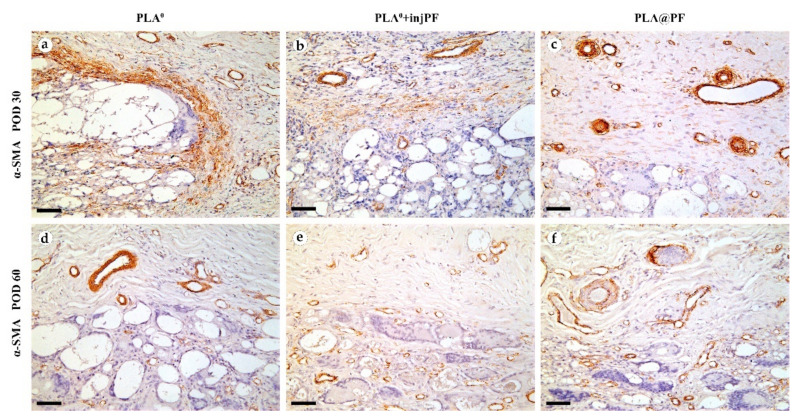
Expression of α-SMA expression in the PIC on POD30 (**a**–**c**) and POD60 (**d**–**f**). Scale bar—50 μm, bright field microscopy. Positive staining is reflected by brown color of DAB, counterstaining with hematoxylin. Columns depict the studied groups (PLA^0^, PLA^0^+injPF and PLA@PF implants).

The expression of α-SMA in the PIC was observable in all groups during the experiment, mostly in activated fibroblasts and in the blood vessels walls (Figure 8). 

On POD30, a thick layer of α-SMA-positive, parallelly oriented myofibroblasts lined the inner part of the peri-implant tissue capsule in PLA^0^ group (Figure 8a). This layer was almost absent in PLA^0^+injPF group. Instead, scattered foci of α-SMA positive fibroblasts were visible around the zone of the ICLT (Figure 7b). In PLA@PF group, the expression of α-SMA was weaker in the capsular fibroblasts, but strong in blood vessels (Figure 8c). However, the peri-implant capsule in this group had a noticeably larger spatial density of blood vessels strongly positive for α-SMA. 

On POD 60, α-SMA expression in all the studied groups was limited mostly to the vascular structures, while it was almost absent in peri-implant tissue fibroblasts (Figure 8d–f).

The number (density per area) of α-SMA-positive blood vessels in peri-implant capsules did not differ between the studied groups on POD30 and POD60, while there was a trend to increase this parameter in PLA^0^+injPF and PLA@PF groups on POD60 vs PLA^0^ on the same time point (Table A3, Appendix A). In PLA^0^ group, the number α-SMA-positive blood vessels in peri-implant capsules decreased statistically significantly from POD30 to POD60, but not in other groups.

**Figure 9:**
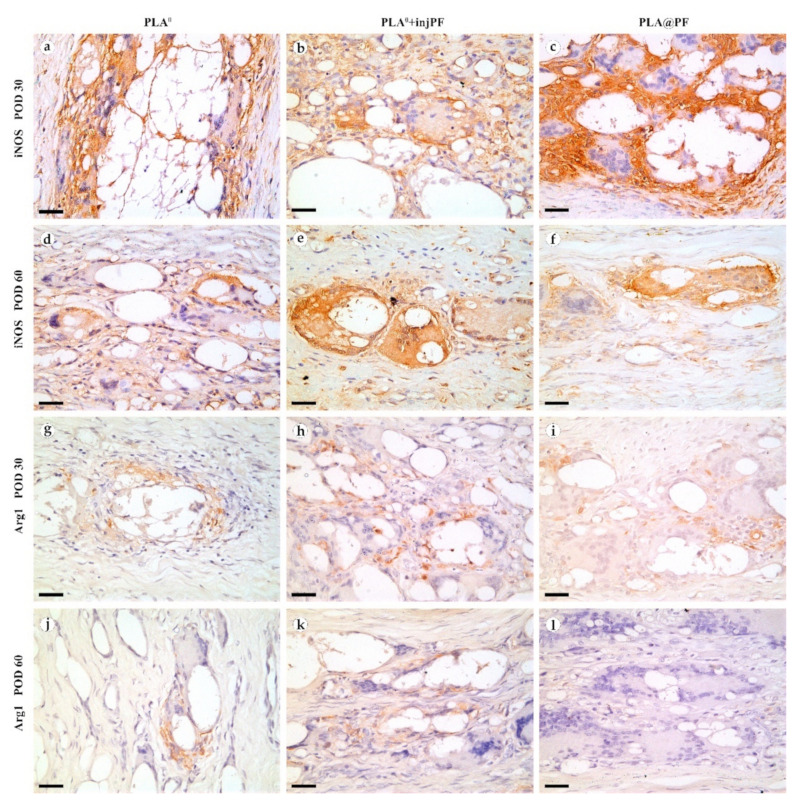
iNOS (**a**–**f**) and Arg1 (**g**–**l**) expression in the PIC on POD30 (**a–c**) and (**g**–**i**) and POD60 (**d**–**f**) and (**j**–**l**). Scale bar—25 μm, bright field microscopy. Positive staining is reflected by brown color of DAB, counterstaining with hematoxylin. Columns depict the studied groups (PLA^0^, PLA^0^+injPF and PLA@PF implants).

**Figure 10:**
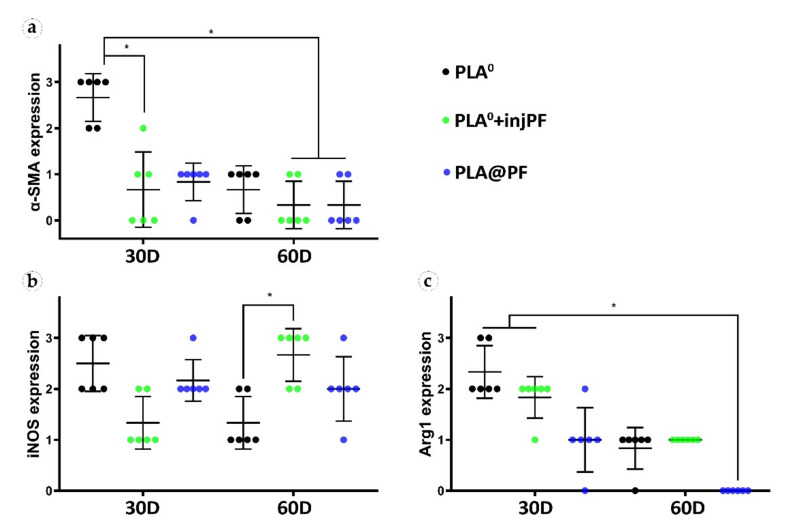
Semi-quantitative scoring analysis of the expression of immunohistochemical markers in PIC examined by the intensity of staining (see Table 1 for the criteria): (**a**) α-SMA, (**b**) iNOS, and (**c**) Arg1. The results are presented as scatterplots, Mean ± SD, * *p* ≤ 0.05.

The expression of iNOS (Figure 9a–f and Figure 10b) was detected in all the groups at both studied time points. The positive staining for this marker was found mostly in the ICLT and, to a much lesser extent, in peri-implant capsules. In particular, the majority of the FBGC were strongly positive for iNOS on POD30. However, a part of FBGC stained negatively for iNOS. 

The statistically significant decrease of α-SMA expression between POD30 and POD60 was observed only in PLA^0^ group (*p* = 0.003), while in PLA^0^+injPF and PLA@PF groups the intensity of the expression of this marker statistically did not change after POD30 (*p* = 0.465 and *p* = 0.093, respectively). The application of PF led to a significant decrease in the expression of α-SMA in fibroblasts in the PLA^0^+injPF and PLA@PF groups on POD30 (Figure 10a). However, on POD60, there were no statistically significant difference in the intensity of α-SMA expression between the studied groups. 

From POD30 to POD60, the expression of iNOS decreased in PLA^0^ group (*p* = 0.011), increased in PLA^0^+injPF group (*p* = 0.007) and was unchanged in PLA@PF group (*p* = 0.598).

There were statistically significant differences in iNOS expression between the studied groups (Figure 10b). On POD30, iNOS expression in PLA^0^ group was higher (*p* = 0.011) and on POD60 it was lower (*p* = 0.007) than in PLA^0^+injPF group. Interestingly, there was no statistically significant difference between the expression of iNOS between PLA^0^ and PLA@PF groups at both time points (*p* = 0.241 for POD30 and *p* = 0.075 for POD60). On POD30, the expression of this marker was also increased in PLA@PF group, in comparison with PLA^0^+injPF group (*p* = 0.018), but did not differ from it on POD60 (*p* = 0.075).

The expression of arginase-1 (Figure 9g–l and Figure 10c) was even more focal to the ICLT and exclusive to macrophages and FBGC. On POD30, the majority of FBGC in PLA^0^ group were Arg1-positive (Figure 9g). The Arg1 expression was weaker in the groups where PF was applied (Figure 9h,i), especially in PLA@PF group. On POD60, single foci containing Arg1-positive FBGC were observed in PLA^0^ and PLA^0^+injPF groups (Figure 9j,k), while in PLA@PF group the PICs were completely Arg1-negative (Figure 9l).

Statistical analysis confirmed that the expression of Arg1 significantly decreased from POD30 to POD60 in all the studied groups (*p* = 0.002 for PLA^0^, *p* = 0.005 for PLA^0^+injPF, and *p* = 0.006 for PLA@PF) (see Figure 10c). The expression for Arg1 did not differ statistically between PLA^0^ and PLA^0^+injPF groups on POD30 (*p* = 0.092) and POD60 (*p* = 0.317). At the same time the expression of this marker was decreased at both studied time points in PLA@PF group, in comparison with PLA^0^ (*p* = 0.007 for POD30 and *p* = 0.005 for POD60) and PLA^0^+injPF (*p* = 0.026 for POD30 and *p* = 0.001 for POD60) groups.

### 3.3. Thermal Analysis

**Figure 11:**
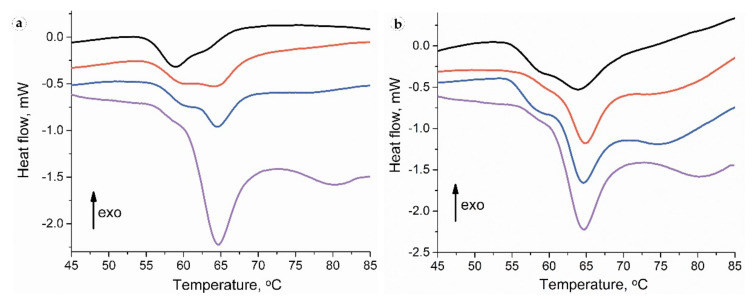
Thermography of peri-implant tissues around PLA^0^ (black), PLA0+injPF (red) and PLA@PF (blue) implants on POD 30 (**a**) and POD60 (**b**). The thermogram of intact ear derma tissue is shown by purple color.

Collagen denaturation DSC thermograms for intact skin samples contained a pronounced peak with a maximum at a temperature of ~64 °C with an adjacent low-temperature shoulder with a peak of ~59 °C and a high-temperature broad and smooth peak (Figure 11). For the full-thickness experimental samples, the collagen denaturation thermograms were within wider limits, and the low-temperature shoulder was transformed into a clear peak with a maximum of ~59 °C. The DSC thermography of separated peri-implant tissues revealed a pronounced low-temperature peak, while the subepidermal areas did not differ from intact samples.

On POD 30, the thermograms of the samples in all studied groups consisted of the low-temperature peak on 40–60% with a maximum ~59 °C (Table 3). Moreover, as it is visible from the Table 3 by the low-temperature peak ratio, in the PLA^0^ implant group, more than a half of the collagen denatured at a temperature <60 °C. The portions of low-temperature peaks in the PLA^0^+injPF and PLA@PF groups also significantly differed from the peaks of the intact dermis but were higher than in PLA^0^ group.

Thermograms of the samples of PLA^0^ group on POD 60 had a notable peak at a temperature of ~59 °C without deconvolution. In the PLA^0^+injPF and PLA@PF groups, this peak had the appearance of a low-temperature shoulder, similar to that of the intact skin.

There were no statistically significant differences of temperature peaks positions between the groups at both studied time points. However, the low-temperature peak ratio statistically significantly differed between the groups on POD30. In particular, it was increased in PLA^0^, PLA^0^+injPF and PLA@PF groups (*p* = 0.004 for all pairs), in comparison with the intact skin derma. There was no statistically significant difference between PLA^0^ and PLA^0^+injPF (*p* = 0.106), and PLA^0^+injPF and PLA@PF groups (*p* = 0.0332). Importantly, the low-temperature peak ratio was significantly decreased in PLA@PF vs. PLA^0^ group (*p* = 0.004). 

### 3.4. Analysis of Correlations

**Figure A4:**
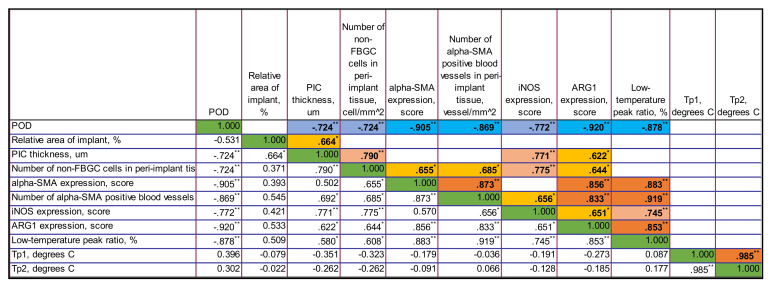
The correlation matrix for PLA^0^ group. Only statistically significant values of Rs are shown in the upper part of the matrix. The color highlights indicate the sign and the strength of the correlation. The tones of blue color show negative correlation, and the ones of orange show the positive correlations. The intensity of the tone reflects the strength of the correlation. The diagonal series of green cells indicates the self-correlation of the same-named variables. ** Correlation is significant at the 0.01 level (2-tailed). * Correlation is significant at the 0.05 level (2-tailed).

**Figure A5:**
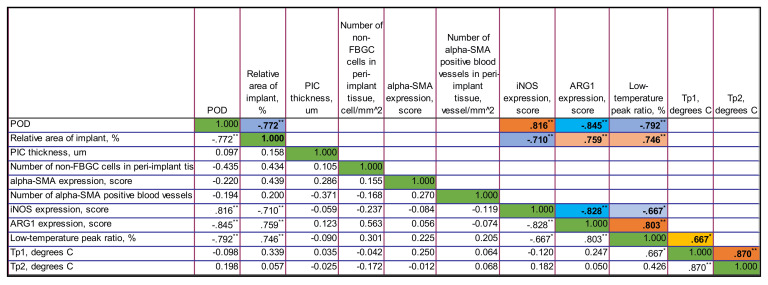
The correlation matrix for PLA^0^+injPF group. Only statistically significant values of Rs are shown in the upper part of the matrix. The color highlights indicate the sign and the strength of the correlation. The tones of blue color show negative correlation, and the ones of orange show the positive correlations. The intensity of the tone reflects the strength of the correlation. The diagonal series of green cells indicates the self-correlation of the same-named variables. ** Correlation is significant at the 0.01 level (2-tailed). * Correlation is significant at the 0.05 level (2-tailed).

**Figure A6:**
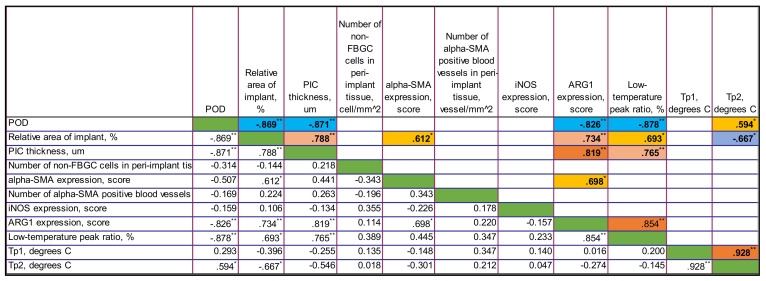
The correlation matrix for PLA@PF group. Only statistically significant values of Rs are shown in the upper part of the matrix. The color highlights indicate the sign and the strength of the correlation. The tones of blue color show negative correlation, and the ones of orange show the positive correlations. The intensity of the tone reflects the strength of the correlation. The diagonal series of green cells indicates the self-correlation of the same-named variables. ** Correlation is significant at the 0.01 level (2-tailed). * Correlation is significant at the 0.05 level (2-tailed).

Statistical analysis of correlations revealed some interesting hidden connections between the studied signatures of FBR and PIF in different groups. The variables included in the correlation matrix are shown in Appendix A (Table A5). The observed statistically significant correlations are considered below and illustrated in Appendix B.1, Figure A4, Figure A5 and Figure A6, in Appendix B. The results of the time-point adjusted correlations and the correlation analysis for the whole data sample are presented in Appendix B (Appendix B.2 and Appendix B.3).

#### 3.4.1. Correlations in PLA^0^ Group

In the PLA^0^ group, there were strong negative statistically significant correlations between several variables and the time after operation. These include the thickness of PIC (Rs = −0.724, *p* = 0.008), number of non-FBGC cells in peri-implant capsule (Rs = −0.724, *p* = 0.008), intensity of α-SMA (Rs = −0.869, *p* < 0.001), expression of iNOS (Rs = 0.772, *p* = 0.003) and Arg1 (Rs = −0.920, *p* < 0.001), and the low-temperature peak ratio in DSC (Rs = −0.878, *p* < 0.001), indicating that these parameters almost linearly decrease between POD30 and POD60.

Relative area of the implant positively correlated with the PIC thickness (Rs = 0.664, *p* = 0.018). 

In turn, the thickness of the PIC positively correlated with the number of non-FBGC cells in the peri-implant capsule (Rs = 0.790, *p* = 0.002), the number (density) of α-SMA-positive blood vessels in peri-implant capsule (Rs = 0.692, *p* = 0.013), the expression of iNOS (Rs = 0.771, *p* = 0.003), the expression of Arg1 (Rs = 0.622, *p* = 0.031) and the low-temperature peak ratio in DSC (Rs = 0.580, *p* = 0.048).

The number of non-FBGC cells in the peri-implant capsule positively correlated with α-SMA expression (Rs = 0.655, *p* = 0.021), the number (density) of α-SMA-positive blood vessels in peri-implant capsule (Rs = 0.685, *p* = 0.014), the expression of iNOS (Rs = 0.775, *p* = 0.003), the expression of Arg1 (Rs = 0.644, *p* = 0.024) and the low-temperature peak ratio in DSC (Rs = 0.608, *p* = 0.036).

The intensity of *α-SMA* expression strongly positively correlated with the number (density) of α-SMA-positive blood vessels in peri-implant capsule (Rs = 0.873, *p* < 0.001), the expression of Arg1 (Rs = 0.856, *p* < 0.001) and the low-temperature peak ratio in DSC (Rs = 0.883, *p* < 0.001). The correlation with the expression of iNOS almost reached the accepted level of statistical significance (Rs = 0.570, *p* = 0.053).

The number (density) of α-SMA-positive blood vessels in peri-implant capsule positively correlated with the expression of iNOS (Rs = 0.656, *p* = 0.021), the expression of Arg1 (Rs = 0.883, *p* = 0.001) and, notably, there was a very strong and highly statistically significant correlation with the low-temperature peak ratio in DSC (Rs = 0.919, *p* < 0.001).

The expression of iNOS positively corelated with the expression of Arg1 (Rs = 0.651, *p* = 0.022) and the low-temperature peak ratio in DSC (Rs = 0.745, *p* = 0.005).

The expression of Arg1 strongly positively correlated with the low-temperature peak ratio in DSC (Rs = 0.853, *p* < 0.001).

#### 3.4.2. Correlations in PLA^0^+injPF Group

In PLA^0^+injPF group, negative statistically significant correlations were found between several variables and the time after operation. These include the relative area of implant (Rs = −0.772, *p* = 0.003), the Arg1 expression (Rs = −0.845, *p* = 0.001) and the low-temperature peak ratio in DSC (Rs = −0.792, *p* = 0.002), indicating that these parameters were decreasing between POD30 and POD60. In contrast to PLA^0^ group, the iNOS expression strongly positively correlated with the time after operation (Rs = 0.816, *p* = 0.001). In addition there were no statistically significant correlations between the time after operation and the thickness of PIC, number of non-FBGC cells in peri-implant capsule, intensity of α-SMA, and other signatures.

The relative area of the implant negatively correlated with the iNOS expression (Rs = −0.710, *p* = 0.010) and positively with Arg1 expression (Rs = 0.759, *p* = 0.004) and the low-temperature peak ratio in DSC (Rs = 0.746, *p* = 0.005).

In contrast to PLA^0^ group, the iNOS expression strongly negatively correlated with Arg1 expression (Rs = −0.828, *p* = 0.001) and the moderate strength negative correlation was observed with the low-temperature peak ratio in DSC (Rs = −0.667, *p* = 0.018).

The expression of Arg1 strongly positively correlated with the low-temperature peak ratio in DSC (Rs = 0.803, *p* = 0.002).

#### 3.4.3. Correlations in PLA@PF Group

In PLA@PF group, there were strong negative statistically significant correlations between the time after operation and the thickness of PIC (Rs = −0.871, *p* < 0.001), the relative area of implant (Rs = −0.869, *p* < 0.001), the expression of Arg1 (Rs = −0.826, *p* = 0.001) and the low-temperature peak ratio in DSC (Rs = 0.878, *p* < 0.001). In contrast to PLA^0^ group, there was no statistically significant connection between the time after operation and the number of non-FBGC cells in peri-implant capsule, intensity of α-SMA and iNOS expression. Notably, the area of the implant in PLA@PF group decreased with time. In PLA@PF implanted animals, not only the area of implant, but also the thickness of PIC decreased with time in comparison to PLA^0^+injPF group.

The relative area of the implant positively correlated with the thickness of PIC (Rs = 0.788, *p* = 0.002), α-SMA (Rs = 0.612, *p* = 0.034) and Arg1 (Rs = 0.734, *p* = 0.007) as well as with the low-temperature peak ratio in DSC (Rs = 0.693, *p* = 0.013). In parallel, it was negatively associated with the temperature peak Tp2 in DSC (Rs = −0.667, *p* = 0.018).

The thickness of PIC positively correlated with the expression of Arg1 (Rs = 0.819, *p* = 0.001) and the low-temperature peak ratio in DSC (Rs = 0.765, *p* = 0.004). 

The expression of α-SMA in PIC positively correlated with the expression of Arg1 (Rs = 0.698, *p* = 0.012).

The expression of Arg1 was strongly positively associated with the low-temperature peak ratio in DSC (Rs = 0.854, *p* < 0.001). 

The temperature peaks Tp1 and Tp2 in DSC were almost linearly interdependent as well (Rs = 0.928, *p* < 0.001).

## 4. Discussion

In the present study, we explored the effects of a new experimental IDDS prototype for the local delivery of PF on FBR and PIF in an ear skin pocket model in rabbits. The animal model used in this work represents a modification of the approach used for the simulation of excessive (hypertrophic) scarring that relies on the ischemic nature of the rabbit ear skin wounds [59,60]. The implants were surgically fixed between skin derma and cartilage plate. Then, the applied methodology allowed to model FBR and PIF in intrinsically pro-fibrotic tissue niche.

The efficiency of an IDDS depends on its drug loading capacity, as well as on the biocompatibility and biodegradation that together contribute to the resulting drug release profile [1,9,17,67,68,69]. In the presented work, a high PF entrapment efficiency of the PLA@PF implants (97.9 ± 13.8% of the original 500 μg PF dose per 0.3 g PLA powder) was achieved by co-foaming of the dry PLA and PF powders in supercritical CO_2_ by the methodology proposed by us earlier [61], that allowed to bypass a problem of poor compatibility of a hydrophobic carrier material (PLA) [58] and a highly hydrophilic drug (PF) [54]. The layer-by-layer laser sintering [62,63] was applied to form the solid and mechanically stable implants. Importantly, in the current research, the regime of the laser treatment was tuned to achieve superficial melting of the edges of the PLA particles, in layers, without overheating of the bulk of the implant structure to prevent damage of the drug molecules. The homogenous distribution of the drug in the PLA carrier following the applied operation was confirmed by the model experiment with EPR spin probe structurally resembling PF. The analysis of the drug release from the sintered PLA@PF implants was performed in vitro in physiological conditions (PBS, 37 °C). This experiment demonstrated that the complete release of PF from PLA@PF implants was prolonged to 80 h, following the initial more rapid release of approximately 50% of the drug during first 20 h. This release time was much longer than the period reported for the different types of PF-loaded ocular lenses (up to 12 h) [70,71].

In this study, we focused on the biomedical aspects of the local delivery of PF by PLA carrier rather than on the development and optimization of the IDDS. In particular, the PF release from the PLA@PF implants in vivo was not directly measured. In addition, it must be noted that the blank PLA^0^ implants and PLA@PF implants had minor, but statistically significant differences in the average size of the particles (where PLA@PF had a finer structure). It is known that this parameter may contribute to the different surface properties and modify the biodegradability of the material. In particular, the polymer implants with smaller size granules show faster degradation [72]. A similar result was demonstrated in our study (see Figure 3b and Table A2 in Appendix A). While these are the limitations of the current work, for the animal ethics considerations, we preferred to narrow the scope to the validation of the principal biological effects of PLA@PF implants first, before the further IDDS development (which is envisaged, in particular, via the adjustments of the scCO_2_ compounds treatment conditions [73]).

The FBR and PIF around PLA@PF and PLA^0^ implants applied alone or in combination with a single topical injection of an equivalent dose of PF were comparatively studied by histopathological methods, DSC and morphometric analysis. As observed on POD30, the FBR developed in all studied groups resulted in the formation of the peri-implant complex (PIC) consisting of the tightly merged resident skin derma, peri-implant capsule and the tissue colocalized with the implant material (ICLT). The ICLT in all the groups contained FBGC that surrounded the implant particles; the fibroblasts, blood capillaries and inflammatory infiltrate with macrophages and lymphocytes. The peri-implant capsules were formed by fibrous connective tissue, where the content of fibroblasts, myofibroblasts and blood vessels, as well as the alignment, density and architectonics of the collagen bundles differed between the studied groups and changed with the time. The structure of the peri-implant capsules in PLA^0^ group was similar to the capsules observed previously around long-term resorbable biomaterials including PLA and polylactic-co-glycolic sutures, textured silicone breast implants [74] and even cochlear implant electrodes [75]. On the other hand, the granulomatous inflammation (emerged in the formation of FBGC) is a typical immune response not only to synthetic biomaterials, but to various xenogeneic structures in the absence of a specific resorption mechanism [76]. Similar responses were demonstrated following the application of chitosan and cellulose films, invasion of parasites and mycobacteria. Based on this, we think that the targeting and disruption of FBR mechanisms is essential to control PIF. 

For the presented IDDS prototype, we chose to use the most known and clinically approved antifibrotic drug, PF, which is a TGF-β1 antagonist with confirmed local effects on fibrosis. The first proposal to apply PF to control FBR was made several years before the FDA approval of the first oral form of PF, Esbriet. In rodents, PF reduced the volume of peri-implant connective tissue and diminished the expression of α-SMA and TGF-beta 1 mRNA [56]. More recently, the same group demonstrated the anti-contraction effect of oral PF on breast implants-related PIF in a clinical trial [77]. Also, local delivery of PF has been proposed to prevent peri-implant capsule formation around the glaucoma drainage device [40]. The antifibrotic effect of PF in these studies was associated with a decrease in the numbers of α-SMA positive cells, which correlates with our results. However, the deeper analysis of the effects of the locally delivered PF has not been provided yet.

Our results revealed several new findings indicating that the PF delivered by a slowly-biodegradable implant can significantly modify the FBR and alleviate or prevent PIF. 

Firstly, we found that the PLA@PF implants were more biodegradable than the PLA^0^ (both, without and with PF injection) as it follows from the results of the measurements of the relative implant area on the histological sections (see Section 3.2.2 and Figure 3b). Interestingly, there were no correlation between the time after operation and the implant area in PLA^0^ group, but in the groups where PF was used the implant area decreased during the experimental period. The delivery of PF by injection did stimulate the biodegradation of the implant, but not to the extent observed in PLA@PF group. It seems reasonable to explain the observed acceleration of the implant degradation by a combinatorial action of PF and the modification of the material properties of the implant after introduction of the PF into the PLA carrier.

Next, it was observed that the thickness of the PIC was similar in the studied groups on POD30, but differed on POD60, when it was dramatically decreased in PLA@PF, in comparison to PLA^0^ and PLA^0^+injPF groups. Importantly, the injected PF did not induce such effect (see Figure 3a and the histological illustrations in Figure 4, Figure 5, Figure 6 and Figure 7). The reliable measurement of the peri-implant capsule thickness that is commonly used for the evaluation of the PIF extent was not possible in the current study because of very interconnected structure of the PIC. Following that, we could not quantitatively proof the effect of PLA@PF on PIF in the same way. Indeed, the thickness of PIC reflected both the intensity of PIF and the degree of the implant biodegradation and remodeling. As the implants degraded faster in PLA@PF group, the volume of the residual implant material that is contributing to the PIC thickness was lower. However, according to the results of correlation analysis, the thickness of PIC positively correlated with the relative implant area only in PLA^0^ and PLA@PF groups, but not in the PF-injected animals. In PLA^0^ group, implant volume did not change with time, while in PF-applied groups it reduced by POD60. Then, only in PLA@PF group the positive correlation of the PIC thickness and the implant area reflected the co-directional change, implying the reduction of the volume of the ICLT and peri-implant capsule and indicating the reduction of PIF.

The study results revealed very interesting differences in FBR and PIF mechanisms between experimental groups. Several positive feedback (self-activation) loops were identified in the tissue of the animals that received the unloaded PLA^0^ implants by the analysis of correlations of histological and immunohistochemical results (see Section 3.4 and Figure A4, Figure A5 and Figure A6 in Appendix B). In particular, we found that the PIC thickness depended on the number of non-FBGC cells (mostly reflecting the intensity of fibroplasia and inflammation in the ICLT), which is, in turn was dependent on iNOS and Arg1 expressions. Overall expression of α-SMA and the number of α-SMA^+^ blood vessels in the PIC were stimulated by iNOS and Arg1. This implies that the ICLT and the inflammatory reaction in this tissue may be the next treatment target to control PIF. In contrast, the majority of these vicious loops were disrupted in PLA@PF group. The thickness of PIC strongly positively correlated only with the Arg1 expression. However, the marker was not expressed in this group on POD60 at all. There was a weaker association between the Arg1 expression and the overall α-SMA signatures, indicating the co-directional changes of these markers. The expression of α-SMA was decreased in the peri-implant tissues treated with PF in comparison to the PLA^0^ group on POD30. This shows that the PF effected myofibroblast transdifferentiation mostly at the initial stage of FBR, which corresponds with the drug release profile observed in vitro. However, PF treatment also had an impact on the innate immunity as reflected by the dynamics of iNOS and Arg1 expression (see Figure 10). Surprisingly, the iNOS expression was stimulated in the animals with the injections of PF on POD60, possibly corresponding with the later onset of the implant biodegradation or an altered pattern of acute inflammation. All in all, the prolonged release of PF from the PLA@PF implants significantly modified the FBR in peri-implant tissues. 

Such an effect is thought to be provided through the key controllers of fibrosis such as macrophages and macrophage-fusion structures, the FBGC [78]. In in vitro experiments, PF inhibited fibrotic activation of fibroblasts on contraction gels [79] and reduced the expression of M2 markers [80]. It is probable, that in our study, most of the drug was delivered to the macrophages/FBGC by the direct contact with implant. As a result, the pro-fibrotic signaling, the myofibroblast transformation and excessive synthesis of collagen were suppressed in PLA@PF group. 

Finally, we revealed a very sensitive measure of the maturation of connective tissue structures using the DSC analysis of the thermal stability of collagen in the peri-implant tissue samples. The obtained observations corroborate with our previous interpretations and conclusions that were done in a scar-modelling experiment on rabbit ears [60]. Briefly, the thermostability of collagen was dependent on amount of covalent cross-links, the interaction of collagen molecules and structural integrity of collagen network. The low-temperature peak (or shoulder) was attributed to the denaturation of the recently synthesized immature collagen characterized by weak crosslinking and poor organization. The main peak corresponded to denaturation of the mature, well organized collagen population stabilized by the crosslinks. In the current study, we revealed the specific connections between the DSC temperature peaks and the low-temperature peak ratio and the histological and immunohistochemical characteristics of the PIC. In particular, the low-temperature peak ratio and its dynamics correlated with the content of α-SMA-positive cells and, especially, with the spatial density of the α-SMA^+^ blood vessels in the PIC. It also positively correlated with the expression of Arg1. We think that the connection between the thermal stability of collagen in the peri-implant tissues and the α-SMA^+^ blood vessels indicated that the synthesis of the collagen with low thermal stability due to low level of cross-linking (as in granulation tissue [81]) occurred in blood vessel cells, implying another potential therapeutic target.

The prototype IDDS proposed in this study is a first step towards development of more sophisticated biodegradable drug delivery systems for subcutaneous implantation and control of PIF. According to the recent reports, a few drug device combination products are currently commercially available. There are several biodegradable polymeric implants approved by FDA for clinical us in the last 5–6 years, including a long-term contraceptive (Nexplanon), a buprenorphine releasing implant (Probuphine) for the treatment of opioid addiction, an absorbable stent for the treatment of coronary atherosclerosis (Abrorb GT1) and an intravitreal dexamethasone releasing implant for the treatment of diabetic macular edema (OZURDEX) [82]. One of the most prominent earlier examples of a clinically used polymer IDDS is Gliadel wafer, a chemotherapy drug-eluting device applied in treatment of glioblastoma [83,84]. There is also a significant market segment taken by non-biodegradable and reservoir-type implants [85,86]. The approval procedure for IDDS depends on the classification criteria, roughly discriminating between the “drug”, “biological agent”, “device” or a “combination product” [82]. Due to the novelty of IDDS, the translation pathways for them are just forming, while the first assessment criteria are also emerging [87]. Considering this information, we presume that the further optimization of the PF-loaded polymer IDDS presented in the current study may be focused on the optimization of the polymer formulation in order to reduce the inflammatory reactions, while maintaining the achieved local anti-fibrotic effect.

## 5. Conclusions

In this study, we demonstrated that a significant amount of pirfenidone can be locally delivered by biodegradable polymer implants, such as a PLA@PF IDDS prototype, for extended local drug release. Locally delivered PF inhibited peri-implant fibrosis via modulation of the foreign body reaction on the polymer implants.

## Figures and Tables

**Table 1:** Criteria of semiquantitative histological scoring of the IHC staining for α-SMA, iNOS and Arg1.

Score	Criteria	
	Expression of α-SMA	Expression of iNOS and Arg1
**0**	Absence of the staining	Absence of staining
**1**	Individual positive cells	Individual positive stained cells
**2**	Thin (≤30 μm) continuous layer of positive cells	≤30% positive stained cells
**3**	Thick (>30 μm) continuous layer of positive cells	>30% positive stained cells

**Table 2:** Thickness of intact skin derma and PIC.

Time Points	Thickness, μm			
	Intact Skin Derma	PIC		
		PLA^0^	PLA^0^+injPF	PLA@PF
**POD30**	302 ± 15	1580 ± 348	1229 ± 402	1249 ± 283
**POD60**		1444 ± 140	1212 ± 163	532 ± 98

**Table 3:** Temperature peaks (Tp) and low-temperature mass portions of thermographs of studied tissues (statistical significance in comparison with the intact tissue: * *p* ≤ 0.05).

Samples		Mean ± St. Deviation		
	Time Point	Tp1, °C	Tp2, °C	The Low-Temperature Peak Ratio, %
Intact derma	N.A.	58.5 ± 1.0	65.6 ± 0.9	15 ± 5.0
PLA^0^	POD30	57.8 ± 1.0	64.7 ± 1.0	60.0 ± 10.0 *
PLA^0^+injPF		58.4 ± 0.8	65.1 ± 1.0	48.0 ±15.0 *
PLA@PF		58.4 ± 1.0	64.9 ± 0.6	40.0 ± 6.0 *
PLA^0^	POD60	58.5 ± 0.7	65.2 ± 0.5	24.0 ± 11.0
PLA^0^+injPF		58.3 ± 1.0	65.5 ± 0.6	23.0 ± 10.0
PLA@PF		58.7 ± 1.0	65.5 ± 0.5	20.0 ± 3.0

## Data Availability

The relevant data generated and (or) analyzed in the current study is available from the corresponding author upon reasonable request.

## Appendix A

**Table A1:** Characterization of the particles’ size in PLA^0^ and PLA@PF implants.

Implant Type	Size, μm					
	Mean ± St. Dev.	95% Confidence Interval for Mean		Median	Min	Max
		Lower Boundary	Upper Boundary			
**PLA^0^**	46.4 ± 29.4	44.9	47.9	36.5	11.3	167.1
**PLA@PF**	40.0 ± 29.9	38.5	41.5	30.0	10.0	170.0

**Table A2:** Dynamics of the relative area of the implants.

Time Point	Group	Relative Area of Implant, %		
		Mean ± St. Dev.	95% Confidence Interval for Mean	
			Lower Boundary	Upper Boundary
**POD30**	**PLA^0^**	104.3 ± 40.8	62.0	147.5
	**PLA^0^+injPF**	92.2 ± 46.8	43.1	141.4
	**PLA@PF**	116.7 ± 50.3	63.9	169.5
**POD60**	**PLA^0^**	64.4 ± 32.2	29.6	99.2
	**PLA^0^+injPF**	33.8 ± 14.9	18.2	49.4
	**PLA@PF**	14.5 ± 3.8	10.5	18.5

**Table A3:** Number of α-SMA-positive blood vessels per mm^2^ in peri-implant capsules.

Time Point	Group	Relative Area of Implant, %		
		Mean ± St. Dev.	95% Confidence Interval for Mean	
			Lower Boundary	Upper Boundary
**POD30**	**PLA^0^**	86.7 ± 20.3	65.3	108.0
	**PLA^0^+injPF**	91.0 ± 43.2	45.7	136.3
	**PLA@PF**	95.0 ± 50.8	41.7	148.3
**POD60**	**PLA^0^**	39.7 ±11.9	27.2	52.1
	**PLA^0^+injPF**	75.0 ± 29.5	44.0	106.0
	**PLA@PF**	78.3 ± 44.9	31.2	125.5

**Table A4:** Number of non-FBGC per mm^2^ in peri-implant capsules.

Time Point	Group	Mean ± St. Dev.	95% Confidence Interval for Mean	
			Lower Boundary	Upper Boundary
**POD30**	**PLA^0^**	5866.7 ± 1268.4	4535.5	7197.8
	**PLA^0^+injPF**	6622.2 ± 3698.6	2740.8	10503.6
	**PLA@PF**	5611.1 ± 1669.3	3859.3	7362.9
**POD60**	**PLA^0^**	3811.1 ± 984.7	2777.7	4844.5
	**PLA^0^+injPF**	3700.0 ± 1261.2	2376.4	5023.6
	**PLA@PF**	4977.8 ± 1576.3	3323.5	6632.0

**Table A5:** Variables included in correlation analysis.

Variable	Type	Measure	Coding of Nominal and Ordinal Variables
Group	Numeric	Nominal	0—intact skin, 1—PLA0, 2—PLA0+injPF, 3—PLA@PF
Was PF applied?	Numeric	Ordinal	0—No, 1—Yes
The time after operation (POD)	Numeric	Ordinal	1—POD30, 2—POD60
Relative area of implant, %	Numeric	Scale	N.A.
Thickness of PIC, um	Numeric	Scale	N.A.
Number of non-FBGC cells in peri-implant tissue, cell/mm^2^	Numeric	Scale	N.A.
Intensity of alpha-SMA expression, score	Numeric	Ordinal	See Table 1
Number of alpha-SMA positive blood vessels in peri-implant tissue, vessel/mm^2^	Numeric	Scale	N.A.
Intensity of iNOS expression, score	Numeric	Ordinal	See Table 1
Intensity of ARG1 expression, score	Numeric	Ordinal	See Table 1
The low temperature peak ratio, %	Numeric	Scale	N.A.
Tp1, °C	Numeric	Scale	N.A.
Tp2, °C	Numeric	Scale	N.A.

## Appendix B

### Appendix B.2. Time Point-Adjusted Correlations

On POD30, in all the groups considered together, the following statistically significant correlations were found.

The application of PF negatively correlated with α-SMA (Rs = −0.678, *p* = 0.002), iNOS (Rs = −0.530, *p* = 0.024) and Arg1 (Rs = −0.766, *p* < 0.001) expression.

The relative area of implant positively correlated with the thickness of PIC (Rs = 0.565, *p* = 0.015). The PIC thickness positively correlated with α-SMA expression (Rs = 0.580, *p* = 0.012) and the low-temperature peak ratio in DSC (Rs = 0.630, *p* = 0.001). The α-SMA expression positively associated with the Arg1 expression (Rs = 0.512, *p* = 0.030). The expression of Arg1 correlated with the low-temperature peak ratio in DSC (Rs = 0.699, *p* = 0.001). The temperature peaks Tp1 and Tp2 in DSC were mutually positively correlated (Rs = 0.930, *p* < 0.001).

On POD60, the application of PF strongly negatively correlated with the relative implant area (Rs = −0.852, *p* < 0.001), and positively correlated with the number of α-SMA positive blood vessels in PIC (Rs = 0.557, *p* = 0.016) and the expression of iNOS (Rs = 0.632, *p* = 0.005).

The relative area of implant positively correlated with the thickness of PIC (Rs = 0.701, *p* < 0.001) and the expression of Arg1 (Rs = 0.758, *p* < 0.001), while at the same time negatively correlated with the number of non-FBGC cells in the PIC (Rs = −0.469, *p* = 0.049). The PIC thickness positively correlated with Arg1 expression (Rs = 0.759, *p* < 0.001) and the low-temperature peak ratio in DSC (Rs = 0.406, *p* = 0.049). There were positive correlations between the DSC parameters: the low-temperature peak ration and Tp1 (Rs = 0.701, *p* < 0.001) and Tp2 (Rs = 0.0686, *p* < 0.001); and between the Tp1 and Tp2 (Rs = 0.907, *p* < 0.001).

### Appendix B.3. Overall Correlations Analysis

In all the groups, along the period of the experiment, the following statistically significant correlations were found.

The time after the operation negatively correlated with the relative implant area (Rs = −0.772, *p* < 0.001), the number of non-FBGC in PIC (Rs = −0.444, *p* = 0.007), the α-SMA expression (Rs = −0.471, *p* = 0.004), the number of α-SMA positive blood vessels in peri-implant area (Rs = −0.399, *p* = 0.016), the expression of Arg1 (Rs = −0.684, *p* < 0.001) and the low-temperature peak ratio in DSC (Rs = −0.525, *p* < 0.001).

The relative area of implant positively correlated with the thickness of PIC (Rs = 0.712, *p* < 0.001), the expression or α-SMA (Rs = 0.512, *p* = 0.001) and Arg1 (Rs = 0.628, *p* < 0.001) and the low-temperature peak ratio in DSC (Rs = 0.657, *p* < 0.001).

The thickness of PIC positively correlated with α-SMA (Rs = 0.515, *p* = 0.001) and Arg1 (Rs = 0.684, *p* < 0.001), the low-temperature peak ratio in DSC (Rs = 0.677, *p* < 0.001) and Tp2 peak (Rs = 0.298, *p* = 0.040). The number of non-FBGC cells in PIC weakly correlated with the low-temperature peak ratio in DSC (Rs = 0.376, *p* = 0.024). The α-SMA expression positively correlated with the expression of Arg1 (Rs = 0.503, *p* = 0.002) and the low-temperature peak ratio in DSC (Rs = 0.557, *p* < 0.001). The number of α-SMA-positive blood vessels in PIC was associated with the low-temperature peak ratio in DSC (Rs = 0.346, *p* = 0.039). The expression of Arg1 correlated with the low-temperature peak ratio in DSC (Rs = 0.790, *p* < 0.001). The temperature peaks Tp1 and Tp2 in DSC were mutually positively correlated (Rs = 0.907, *p* < 0.001).
